# Selected cell wall-associated components in plant defense responses against microbial pathogens

**DOI:** 10.3389/fpls.2026.1896272

**Published:** 2026-07-14

**Authors:** Piotr Rusin, Edmund Kozieł, César Escalante, Katarzyna Otulak-Kozieł

**Affiliations:** 1 Institute of Biology, Department of Botany and Plant Physiology, Warsaw University of Life Sciences-SGGW, Warsaw, Poland; 2 Department of Botany and Plant Pathology, Purdue University, West Lafayette, IN, United States

**Keywords:** cell wall-associated non-enzymatic proteins, cellulases, expansins, extensins, glycosyltransferases, host defense reaction, pectinases, pectin-modifying enzymes

## Abstract

In the field of plant-microbe interactions, numerous cellular components of plants are known to play a critical role in interactions with pathogens. Nevertheless, a comprehensive understanding of all aspects of these interactions is lacking. Significant advancements have been made regarding the involvement of cell wall compounds in the plants’ overall response against biotic threats, and the findings indicate that certain molecules directly or indirectly influence cell wall alterations. The plant cell wall plays a vital role in providing a dynamic response against pathogenic microorganisms. Several groups of cellular components substantially affect cell wall structure, including enzymes involved in the synthesis and degradation of cellulose and hemicellulose, enzymes related to pectin modification, cell wall-associated non-enzymatic proteins, and pathogenesis-related proteins. These components contribute to the development of effective resistance, which can be manifested, for example, as a hypersensitive response. Conversely, the same components can cause vulnerability in different pathosystems, facilitating the growth of pathogens. The present review sums up the roles of enzymatic and non-enzymatic cell wall components in the defense response to important plant pathogens.

## 1 Introduction

The cell wall (CW) is the primary barrier against pathogen infection; it serves not only as a mechanical barrier between the intracellular and extracellular environments but also plays a crucial role in modulating antimicrobial responses in plants (Bacete et al., 2018; Pinto et al., 2025; Gross et al., 2025). CW, a component of the plant apoplast, is a nutrient-rich environment that makes it a suitable habitat for the growth of different microorganisms (Underwood, 2012; Fatima and Senthil-Kumar, 2015). Consequently, plants have evolved immune responses mediated by signals from cellular and extracellular receptors, leading to apoplastic immunity that can be highly efficient in providing resistance against biotic stresses (Doehlemann and Hemetsberger, 2013; Zhang et al., 2020; Wang et al., 2017). In this process, the critical factors are reactive oxygen species (ROS) production and accumulation of antioxidant enzymes regulating ROS concentration (Farvardin et al., 2020). Elevated ROS levels significantly impact saccharide synthesis, resulting in the local deposition of structural CW compounds. These changes are often accompanied by cell wall alkalization, which subsequently enhances CW rigidity (Munzert and Engelsdorf, 2025). Moreover, plants have developed mechanisms to maintain CW integrity, which involve various kinase families that remodel themselves to provide CW reinforcement at the infection site, as an apparent defense response to pathological microorganisms (Wan et al., 2021). Therefore, plant’s general reaction leading to CW rearrangement is initiated through two parallel processes, namely pattern-triggered immunity (PTI) and effector-triggered immunity (ETI), which represent two fundamental defense mechanisms against different microorganisms (Banerjee et al., 2025) such as bacteria (Zhang and Zhou, 2010), viruses (Ivanov et al., 2023), and fungi (Guo and Cheng, 2022). PTI requires the presence of specific molecules associated with pathogen infection, referred to as pathogen-associated molecular patterns (PAMPs) (Zipfel and Robatzek, 2010). PAMPs are recognized by proteins located in the plasma membrane; these proteins are known as pattern recognition receptors (PRRs) and possess extracellular domains with leucine-rich repeats (LRRs). PRRs like FLAGELLIN SENSING 2 (FLS2) and EF-Tu RECEPTOR (EFR), are mainly transmembrane receptor-like kinases (RLKs) or receptor-like proteins (RLPs) (Ryu et al., 2025). The one of RLPs: RLP44 interacts with components of the brassinosteroid signaling pathway during antimicrobial response (Kohorn, 2015) and controls plant response to abnormal increased methylated pectin (mPectin) presence at the cell wall (Huerta et al., 2023). In general, PRRs are a part of surveillance system able to detect PAMPs along with self-derived damage-associated molecular patterns (DAMPs) (Boller and Felix, 2009; Newman et al., 2013; Zipfel, 2014). Furthermore, cellular calcium signatures modulate PAMPs and DAMPs pathogen recognition. However, the exact nature of Ca^2+^ signaling during pathogen defense inductions is rather limited to mechanistic understanding of how various calcium signatures affect gene expression and defense response (Seybold et al., 2014; Zhang et al., 2025). Aslam et al. (2008) and Ranf et al. (2011) postulated apoplastic origin of PAMP-induced Ca^2+^ influx crucial for anti-pathogen response. On the other hand, Ma et al. (2012) outlined importance of intracellular Ca^2+^ accumulation and storage. Nevertheless during PAMP perception, PRRs associate or dis-associate with specific partner proteins provide point of start for downstream for transduction of defense signaling along with rapid burst of Ca^2+^and reactive oxygen species (ROS), activation of kinase cascades (via PRRs), Ca^2+^-dependent protein kinases (CDPKs) and mitogen-activated protein kinases (MAPKs) (Chinchilla et al., 2007; Boller and Felix, 2009; Sun et al., 2013; Macho and Zipfel, 2014). PRRs functions are essential for the PTI. Mutations in *FLS2* (loss-of-function) impair *Arabidopsis thaliana* resistance against *Pseudomonas syringae* pv. tomato (Pst) DC3000 bacteria (Zipfel et al., 2004). Whereas, Arabidopsis *efr* mutants showed increased susceptibility to *Agrobacterium tumefaciens* (Zipfel et al., 2006). It was noticed that the *cerk1* mutants display increased susceptibility to fungal pathogens (Miya et al., 2007; Wan et al., 2008), while *pepr1* and *pepr2* Arabidopsis mutants has increased susceptibility to Pst DC3000, *Botrytis cinerea*, and *Colletotrichum higginsianum* compared to WT plants (Ma et al., 2012; Liu et al., 2013; Ross et al., 2014). Nevertheless, in PTI, one of the earliest effects of pathogen recognition by PRRs is the successive activation of mitogen-activated protein kinases (MAPKs) that stimulate the activity of different proteins associated with subsequent biosynthesis of salicylic acid (SA), jasmonic acid (JA), ethylene, phytoalexins, nitric oxide (NO), and ROS (Meng and Zhang, 2013).

On the other hand, we have ETI associated with the activation of subcellular nucleotide-binding oligomerization domain-like receptors (NLR) proteins, which comprise a nucleotide-binding site (NBS), an LRR fragment at the C-terminus, and different domains located at the N-terminus (Dalio et al., 2021; Yuan et al., 2021). NLRs mediate interactions with pathogen effectors, the so-called avirulence factors (Avr). Pathogens counteract the defense response by producing various virulence factors, inducing effector-triggered susceptibility (ETS). This observation suggests that the plant defense response should be treated as plant-pathogen signaling crosstalk (Ma et al., 2018).

**Figure 1:**
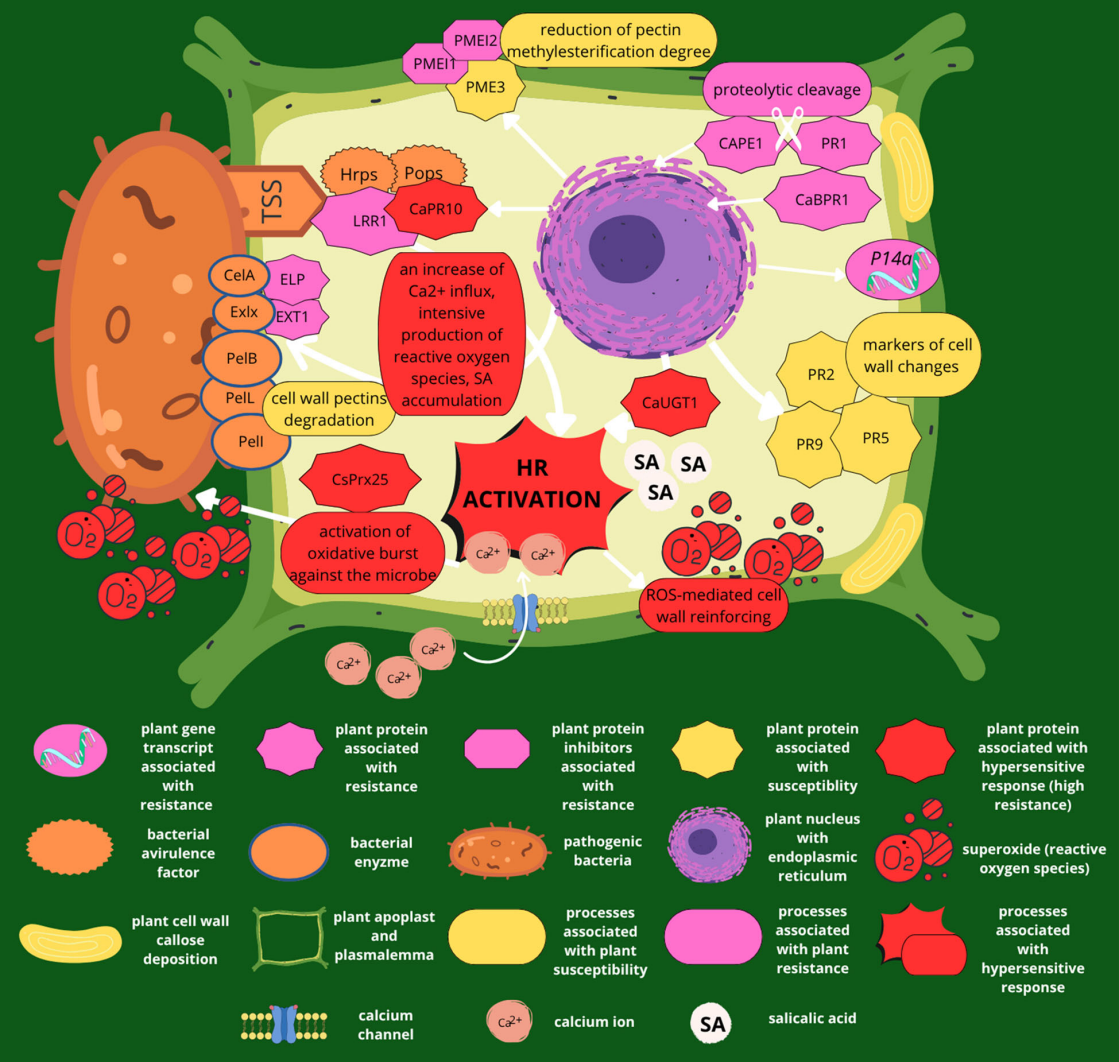
Overview of selected bacterial and plant cellular compounds (PRs, expansins, extensins, and polysaccharide modification-related enzymes) influencing plant-pathogenic bacteria interactions (own work inspired by the scheme from Nguyen et al., 2021). Plant defensive reactions can be altered by various endogenous plant and bacterial proteins. Pathogenic bacteria can secrete proteins via various types of secretion systems (TSS) (Setti et al., 2014). Some of them are so-called avirulence factors (Avr), including *Pseudomonas* outer proteins (Pops) (Li et al., 2010) and harpins (Hrps) (Choi et al., 2013), which trigger the hypersensitive response (HR). The recognition process of Pops and Hrps is managed by nucleotide-binding oligomerization domain-like receptors (NLR) (Yuan et al., 2021), especially by leucine-rich repeat protein 1 (LRR1), which requires *Capsicum annum* pathogenesis-related protein 10 (CaPR10) (Choi et al., 2012) for HR signal transmission by kinase cascades (Swaminathan et al., 2022). *Capsicum annum* UDP-glucosyltransferase 1 (CaUGT1) is also an HR-specific protein involved in the signaling cascades during plant-bacteria interactions (Lee et al., 2009). HR activation involves calcium influx, increased salicylic acid (SA) accumulation, and intensive production of reactive oxygen species (ROS) (Meng and Zhang, 2013). Elevated ROS levels in the apoplast can reinforce the plant cell wall (CW) by participating in the cross-linking of macromolecules (Hamann, 2012). ROS in plant-bacteria interactions can be increased by plant *Citrus sinensis* peroxidase 25 (CsPrx25), classified as pathogenesis-related 9 (PR-9) protein (Li et al., 2020). Defensive process also involves the induction of plant proteins and/or transcripts. For example, *Capsicum annum* basic pathogenesis-related protein 1 (CaBPR1) can influence the expression of genes involved in the defense response, including those encoding PR enzymes (Sarowar et al., 2005). However, during plant-bacteria interactions, CW localization of pathogenesis-related proteins (PR3, PR5, and PR9) can serve as a marker of plant CW changes (Dahal et al., 2010). The signal that alters plant expression patterns comes from the release of CAP-derived peptide 1 (CAPE1) from PR1 proteins after proteolytic cleavage (marked by white scissors) and relocation of CAPE1 to the nucleus (Chen et al., 2014). Another *P14a* gene encoding the P14a, classified as PR1 protein, is expressed in *Solanum lycopersicum*.The P14a protein localizes in the cells’ periphery at the infection site and reduces the spread of *Pseudomonas syringae* infection (Lincoln et al., 2018). Pathogenic bacteria produce enzymes decreasing the integrity of the plant CW eg. bacterial expansin (Exlx) (Rocha et al., 2020), cellulase (CelA) (Hwang et al., 2019), and pectinases (PelB, PelL, PelI) (Fagard et al., 2007). Plants can produce cell wall-associated proteins, eg, extensin-like protein (ELP) (Balaji and Smart, 2012) and extensin 1 (EXT1) (Wei and Shirsat, 2006) that provide cell wall fortification reinforcement at the infection site. Bacteria can also induce the production of pectin methylesterase 3 (PME3), a plant enzyme that reduces the degree of methylesterification of plant CW pectins. PME3 contributes to plant cell wall degradation and facilitate bacterial infection (Raiola et al., 2011). In response, plants can induce the localization of pectin methylesterase inhibitor 1 (PMEI1) and pectin methylesterase inhibitor 2 (PMEI2), reducing PME3 activity (Raiola et al., 2011).

**Figure 2:**
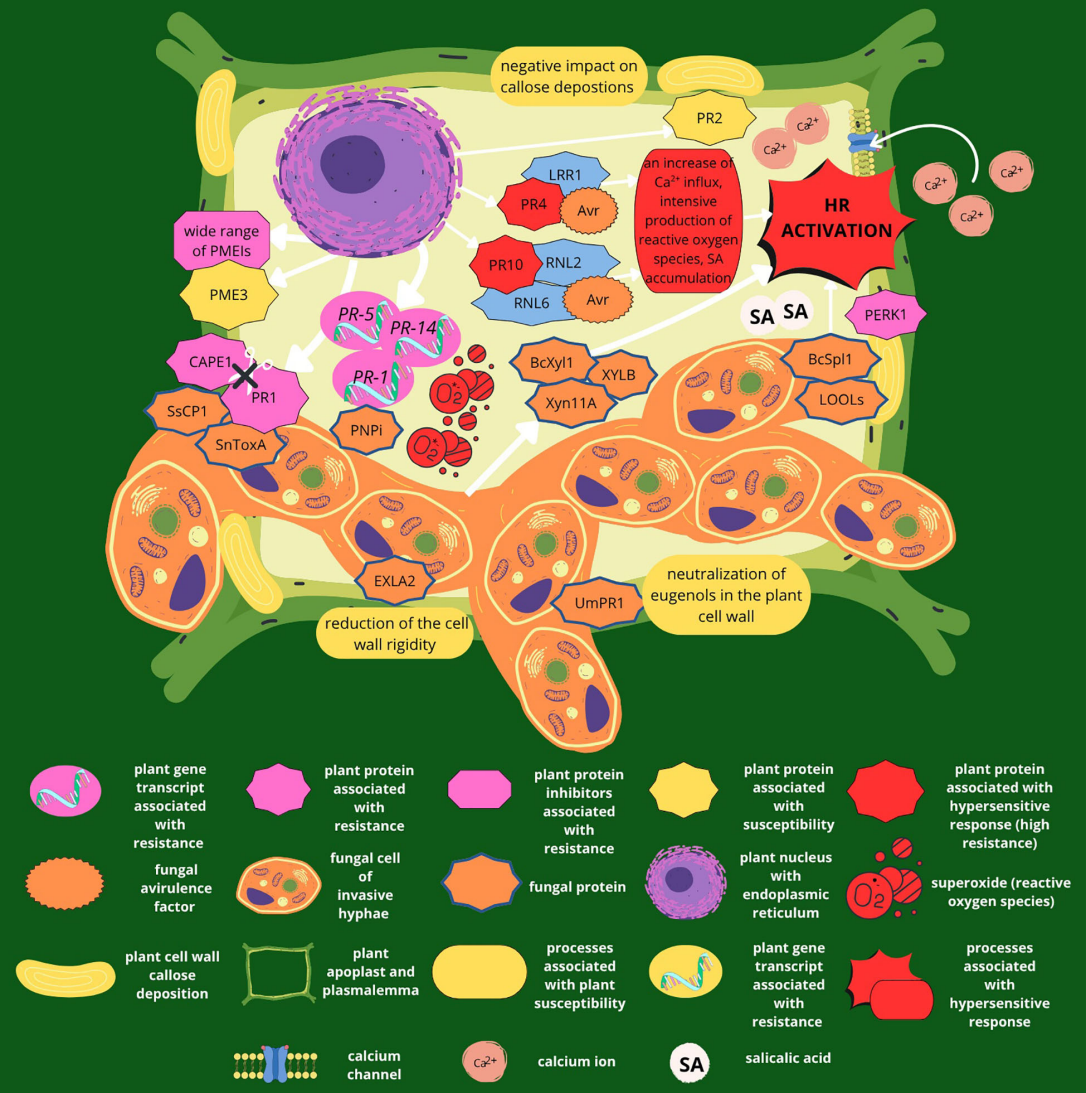
Overview of selected fungal and plant cellular compounds (PRs, expansins, extensins, and polysaccharide modification-associated enzymes) influencing plant-pathogenic fungi interactions (own work, inspired by the scheme from Nguyen et al., 2021). Pathogenic fungi form multicellular hyphae during interactions with plants, allowing them to breach the plant cell wall (CW) and colonize plant cells (Cruz-Mireles et al., 2021). Pathogenic fungi use different proteins to facilitate colonization. The group of 3 proteins from different species: *Sclerotinia sclerotiorum* cerato-platanin protein 1 (SsCP1) and *Stagonospora nodorum* ToxA (SnToxA) render plant susceptibility to fungal infections by physical interaction with pathogenesis-related protein 1 (PR1), which probably restricts CAP-derived peptide 1 (CAPE1) release (marked with a crossed-out scissors), whereas *Puccinia nonexpressor PR 1* (*NPR1*) genes interactor (PNPi) downregulates the *PR-1* expression (Han et al., 2023). *PR-1* is co-expressed with *PR-5*, encoding thaumatin-like proteins, and *PR-14*, encoding lipid transfer proteins, and it enhances resistance to fungal pathogens by increasing ROS production (Han et al., 2023). Some fungi can also produce their own PR1 proteins, such as *Ustilago maydis* PR1 (UmPR1), which neutralize antimicrobial eugenols in the plant CW (Lin et al., 2023). However, PR-2 can be a susceptibility factor in plant-fungi interactions, as β-1,3-glucanase degrades CW callose reinforcements (Oide et al., 2013). Some fungi also produce loosenin-like proteins (LOOLs), which can disrupt interactions between structural polysaccharides in the plant cell wall (Monschein et al., 2023). *Botrytis cinerea* cerato-platanin (BcSpl1) can cause rapid hypersensitive reaction (HR) necrosis, which increases the plant’s susceptibility to necrotrophic pathogens (Frías et al., 2011). During plant infections with pathogenic fungi, HR activation is mediated by the interaction of pathogenesis-related 4 proteins (PR4) with leucine-rich repeat protein 1 (LRR1) (Hwang et al., 2014) and pathogenesis-related 10 (PR10) with nucleotide-binding oligomerization domain-like receptors (RNL2 and RNL6) and fungal avirulence factors (Avr) (Zhang et al., 2021). HR activation involves calcium influx, increased salicylic acid (SA) accumulation, and intensive production of reactive oxygen species (ROS) (Meng and Zhang, 2013). Elevated ROS levels in the apoplast can reinforce the plant cell wall by participating in the cross-linking of macromolecules (Hamann, 2012). Moreover, PERK1 is likely a regulator of resistant defensive plant responses by participating in signal transduction of CW damage to other kinases, as in the *Brassica napus- Sclerotinia sclerotiorum* interaction (Silva and Goring, 2002). Besides, fungal presence in plants stimulates the production of pectin methylesterase 3 (PME3) that decreases the methylesterification degree of plant CW pectins, which contributes to the plant CW degradation (Raiola et al., 2011). In response, plants produce multiple plant pectin methylesterase inhibitors that reduce PME3 activity (PMEIs) (Lionetti et al., 2017; Wang et al., 2022).

**Figure 3:**
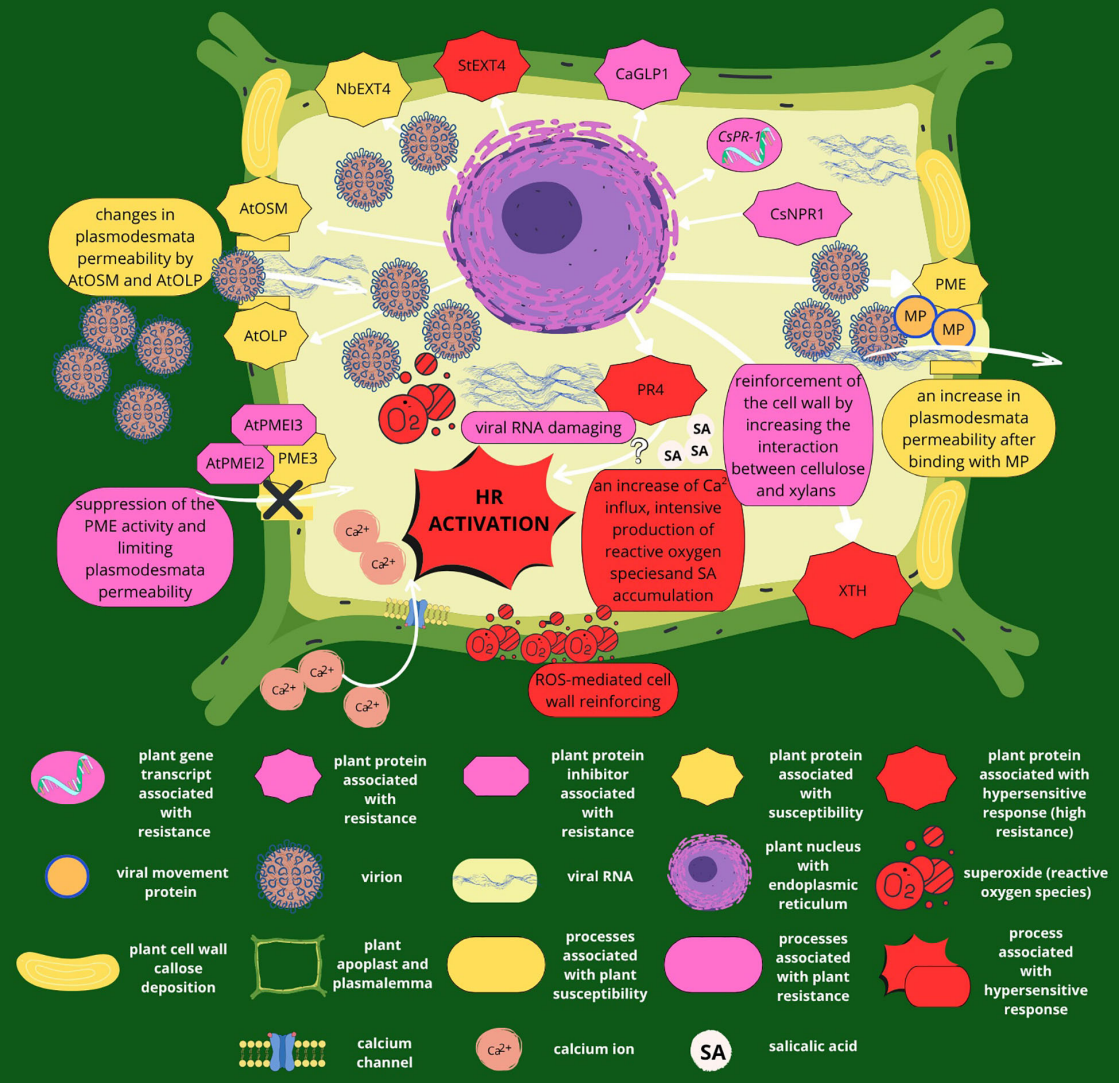
Overview of selected plant cellular compounds (PRs, expansins, extensins, and polysaccharide modification-associated enzymes) influencing plant-virus interactions (own work inspired by the scheme from Nguyen et al., 2021). Viruses can enter plant cells by cell wall (CW) damage and then through plasmodesmata (Kumar et al., 2015). Permeability of plasmodesmata is possibly increased by two *Arabidopsis* proteins from pathogenesis-related 5 (PR-5) family: *Arabidopsis thaliana* osmotin protein 34 (AtOSM34) and *Arabidopsis thaliana* osmotin-like protein (AtOLP) (He et al., 2025). Plasmodesmata permeability can also be increased by direct interactions between pectin methylesterases (PMEs) and viral movement proteins (MPs), facilitating the transport of virions and viral genetic material to neighboring plant cells from the infection site (Kumar and Dasgupta, 2021). However, interaction between PMEs and MPs can be disturbed when PMEs are inhibited by pectin methylesterase inhibitors (PMEI2 and PMEI3), which limits the transmission of the virus from cell to cell (indicated by Otulak-Kozieł et al., 2024). Viral presence in plant cells induces the localization of proteins involved in the resistance response. *Capsicum annuum* germin-like protein 1 (CaGLP1), classified as PR-16 protein, localizes in the cell wall during viral infection and participates in cell wall remodeling (Park et al., 2004). *Cymbidium* sp. nonexpressor of pathogenesis-related 1 (CsNPR1) is relocated during a viral infection from cytoplasm to nucleus and is responsible for stimulating the plant’s defense response and expression of *CsPR-1* as a marker of some viral infections (Ren et al., 2020). PR4 proteins are also induced and possess ribonuclease activity that can degrade viral RNA and may hypothetically participate in HR activation (indicated by a question mark) (Guevara-Morato et al., 2010). HR activation involves calcium influx, increased salicylic acid (SA) accumulation, and intensive production of reactive oxygen species (ROS) (Meng and Zhang, 2013). Increased ROS levels in the apoplast can reinforce the plant cell wall by cross-linking of macromolecules (Hamann, 2012). *Solanum tuberosum* XTH-Xet5 (Xet5 xyloglucan xyloglucosyl transferase) influenced on interactions between cellulose and hemicelluloses in plant CW (Otulak-Kozieł et al., 2018). Moreover, *Solanum tuberosum* extensin 4 (StEXT4) participates in reinforcing the cell wall by creating a network of cross-linked proteins (Otulak-Kozieł et al., 2020).

Both PTI and ETI response are crucial in plant host defense system (Li and Weigel, 2021) as well as carbohydrate active enzymes (CAZymes). The CAZymes is a broad group of enzymes involved in the synthesis, modification and breakdown of different types of carbohydrates (Garron and Henrissat, 2019). This proteins are able to enter into host-pathogen battleground as part of response and actively modification CW structure and generation of oligosaccharide fragments needed for recognition and induction of immune response. Among them are also cellulases, pectinases and xylanases, play a unique role in CW modification. These enzymes are adapted for direct interactions with the structural polysaccharides of the CW (Garron and Henrissat, 2019), expansins, extensins, PRs, and enzymes involved in CW pectin modification. These molecules participate in different types of plant interactions, including the HR, with viral (Kozieł et al., 2021), fungal (Lorrai and Ferrari, 2021) and bacterial (Cosgrove, 2015) pathogens. The most interesting reports mentioned the role of the proteins regulating CW structure during resistant and susceptible plant reaction against pathogens are summarized in **Table 1**. Although there are more publications unmentioned in the text, threatening resistance factors against pathogenic bacteria, viruses and fungi, which were listed in **Supplementary Table 1**. However, not all proteins or enzymes play a role in enhancing resistance. Plants during the evolution create the so-called cell wall-degrading proteins (CWDPs), which partially degrade the CW to enabled physiological processes like fruit ripening. Moreover, cell wall loosening is crucial for seed release (Cantu et al., 2008). It was noticed that some necrotrophic pathogens learn to control expression of genes encoded CWDPs to successfully infect fruits. CWDPs are secreted into CW during specific stages of growing season of plant or fruit development as well as disrupt the function and/or localization of molecules with an important role in antimicrobial response. This easily affects increasing plant susceptibility to pathogens (Cantu et al., 2008) and indicates the complexity of CW remodeling processes upon anti- and pro-pathogen reaction. The aim of this review is to outline and sum up the role of cellulose synthases, cellulases, pectinases, xylanases, xylan-modifying enzymes, pectin-associated enzymes, expansins, extensins, and PR proteins involved in the CW reorganization process, indicating whether they favor resistant or susceptible plant interactions with pathogenic bacteria (**Figure 1**), fungi (**Figure 2**), and viruses (**Figure 3**).

**Table 1:** The summary of reports indicating CW-associated components and/or their genes as susceptibility or resistance factors in certain pathosystems.

CW component(s) and/or gene(s)	The effect(s) on plant’s cell wall	Molecular processes participating in the plant response associated with the plant cell wall component	Associated pathosystem(s)	Reference(s)
Resistance factors				
Celulose synthases (CESA4/IRX5, CESA7/IRX3, and CESA8/IRX1)	Limited secondary-cell wall formation associated with loss of function of CESA 4,7 and 8 genes altered xylem integrity.	Accumulation of antimicrobial secondary metabolites	*Arabidopsis thaliana- Ralstonia solanacearum; Arabidopsis thaliana- Plectosphaerella cucumerina*	Hernández-Blanco et al., 2007; Ramírez et al., 2011
Substitution enzyme of xylan (*GhGUX5*)	Increased expression of *GhGUX5* altered gene expression in the phenylalanine ammonia-lyase pathway, resulting in increased CW lignin content.	Accumulation of plant hormones	*Gossypium hirsutum* cv. *Zhongzhimian 2- Verticillium dahliae*	Zhang et al., 2023
Sugar-dependent glycosyltransferases (UGT73C3 and UGT73C4)	UGT73C3 and UGT73C4 promote the formation of pinoresinol diglucoside (PDG), which increases cell wall callose deposition and levels of ROS, influencing CW reorganization.	Glycosylation of lignans	*Arabidopsis thaliana- Pseudomonas syringae pv. Tomato (Pst)* DC3000	Zhao et al., 2025
Extensin (EXT4)	Cell wall reinforcement provided by HRGPs, especially during HR.	Subcellular localization and macromolecular cross-linking	*Solanum tuberosum* cv. *Sárpo Mira- Potyvirus yituberosi* (PVY^NTN^)	Otulak-Kozieł et al., 2020
Extensin-like protein (ELP)	Cell wall reinforcement provided by ELP.	Gene overexpression, subcellular localization, and macromolecular cross-linking	*Solanum lycopersicum- Clavibacter michiganensis subsp. Michiganensis (Cmm)*	Balaji and Smart, 2012
Proline extensin-like receptor kinase 1 (PERK1)	PERK1 provides sensing of CW mechanical damage and supports adherence of CW to plasmalemma.	Subcellular localization	*Brassica napus- Sclerotinia sclerotiorum*	Silva and Goring, 2002
Pectin methylesterase inhibitor (GhPMEI3)	GhPMEI3 inhibited the activity of GhPME2 and GhPME31, which prevented CW pectins from intensive demethylation and degradation.	Enzyme inhibition and DAMP release kinetics	*Gossypium hirsutum- Verticillium dahliae*	Liu et al., 2018
Pectin methylesterase inhibitor (AtPMEI1)	PMEI1 inhibited the activity of PME3, which prevented CW pectins from intensive demetyloesterification and degradation.	Gene overexpression and enzyme inhibition	*Arabidopis thaliana - Botrytis cinerea*	Lionetti et al., 2007
Actinidia chinensis pectin methylesterase inhibitor (AcPMEI)	AcPMEI inhibited the general activity of PME that prevented CW pectins from intensive demetyloesterification and degradation.	Gene overexpression and enzyme inhibition	*Nicotiana tabacum- Tobamovirus tabaci* (TMV)	Lionetti et al., 2014
Pectin methylesterase inhibitors (AtPMEI2 and AtPMEI3)	AtPMEI2 and AtPMEI3 inhibited the activity of PME3 that prevented CW pectins from intensive demetyloesterfication and consequently degradation.	Enzyme inhibition	*Arabidopis thaliana - Potyvirus rapae* (TuMV)	Otulak-Kozieł et al., 2024
Rice xylanase inhibitor (*RIXI*)	RIXI overexpression increased plant resistance to fungal infection by inhibiting harmful xylanases.	Gene overexpression and enzyme inhibition	*Oryza sativa* cv. *Nipponbare- Magnaporthe oryzae*	Hou et al., 2015
Triticum aestivum xylanase inhibitor-I (*TAXI-I*)	TAXI-I overexpression reduced the occurrence of necrosis and cell wall degradation caused by fungal xylanase.	Gene overexpression and enzyme inhibition	*Arabidopsis thaliana- Botrytis cinerea*	Tundo et al., 2020
TAXI-III and xylanase-inhibiting protein (XIP-I)	Proteins reduced the ability of fungal xylanases to cause necrosis and xylanase activity.	Gene overexpression and enzyme inhibition	*Triticum aestivum- Fusarium graminearum*	Tundo et al., 2015
Xyloglucosyl transferase (XTH-Xet5)	XTH-Xet5 is especially induced during HR and reinforces plant CW by rearranging cellulose and hemicellulose interactions.	Subcellular localization	*Solanum tuberosum* cv. *Sárpo Mira*- *Potyvirus yituberosi* (PVY^NTN^)	Otulak-Kozieł et al., 2018
Susceptibility factors				
Celulose synthase (*TaCESA7*)	Upregulation of TaCESA7 expression indicates a restriction in lignin biosynthesis.	Phenylpropanoid biosynthesis and DAMP release kinetics	*Triticum aestivum- Puccinia striiformis f. Sp. Ritici*	Zhang et al., 2025b
Cellulose synthases (*CESA3* and *CESA6*)	Upregulated expression of CESA3 (je5) and CESA6 (prc1-1) caused a trade-off between cellulose (increased) and callose (decreased) CW content.	Bacterial quorum sensing attenuating PTI	*Arabidopsis thaliana- Xanthomonas campestris*	Liu et al., 2024
Endo-β-1,4-glucanase (*LrCel1*)	*LrCel1* overexpression caused restriction in cellulose, lignin and callose biosynthesis.	Gene overexpression and accumulation of plant hormones	*Nicotiana benthamiana-Fusarium oxysporum; Nicotiana tabacum-Fusarium oxysporum*	Chen et al., 2025
Tomato wound-induced 1 (*Twi1*)	The expression of *Twi1* restricted possible ROS-dependent CW remodeling during HR by intensive accumulation of antioxidative flavonoids.	Metabolism of flavonoids	*Solanum lycopersicum- Orthotospovirus tomatomaculae* (TSWV)	Campos et al., 2019
Expansin (EXPA4)	Accumulation of EXPA4 disrupts the arrangement between cellulose and hemicellulose in CW, making the plant vulnerable to wounding and infections.	Gene overexpression	*Nicotiana benthamiana- Tobamovirus tabaci* (TMV)	Chen et al., 2018
Pectin methylesterase (AtPME3)	Increased activity of PME3 caused intensive demethyloesteryfication of CW pectins which favored CW decomposition.	Subcellular localization and pectin demethylesterification	*Arabidopsis thaliana- Pectobacterium carotovorum*	Raiola et al., 2011

## 2 Involvement of enzymes associated with the modification and degradation of cellulose, hemicellulose and pectins in CW rearrangement induced by plant microbial pathogens

### 2.1 Plant cellulose synthase complexes

Cellulose has a vital role in plant CW apoplastic immunity. Cellulose chains are produced by cellulose synthase complexes (CSCs) located in the plasmalemma and arranged in bundles known as microfibrils, which contain crystalline and amorphous regions (Kulasinski et al., 2014). The proportion of various organized regions of microfibrils influences CW rigidity and accessibility of enzymes to cellulose (Lorrai and Ferrari, 2021). CSCs are composed of multiple cellulose synthases (CESAs), which influence developmental processes and responses to biotic stresses by forming primary and secondary CWs (Wan et al., 2021). *Arabidopsis thaliana* possesses 10 CESA proteins, including those involved in primary CW formation (CESA1, CESA3, and CESA6) and a group associated with secondary CW formation (CESA4, CESA7, and CESA8) (Hill et al., 2014). The functionality of the remaining proteins (CESA2, CESA5, CESA9, and CESA10) is complicated (Desprez et al., 2007; Chu et al., 2007). *CESA2*, *CESA5* and *CESA9* are expressed in tissues with rapidly dividing cells like root or hypocotyl during elongation (Persson et al., 2007) but also in plant embryo (Beeckman et al., 2002). However, Beeckman et al. (2002) excluded importance of *CESA5* in embryo base on level of expression. The *CESA9* is also highly expressed in anthers and pollen, whereas *CESA5* is observed into light-grown tissues of leaves and hypocotyl (Persson et al., 2007). The Griffiths et al. (2015) revealed the role of *CESA5* and *CESA10* in formation of seed mucilage adherence to seed coat epidermal cells. Moreover, an impairment in secondary CW formation elevates resistance to pathogens, as observed for *A. thaliana* mutants deficient in CSC subunits, namely (**Table 1**) CESA4/IRREGULAR XYLEM5-IRX5, CesA7/IRX3, CesA8/IRX1, and inoculated with *Ralstonia solanacearum* and *Plectosphaerella cucumerina* (Hernández-Blanco et al., 2007; Ramírez et al., 2011). Furthermore, *cesa4*, *cesa7*, *cesa8* mutants characterized by increased resistance via altered xylem integrity which created changes in: xylem water transport, distribution of ABA and vascular connectivity. This amount of xylem changes disturbed and mitigated spreading of pathogens in host plant (Hernández-Blanco et al., 2007; Ramírez et al., 2011). Conversely, significant upregulation of *TaCESA7* from *Triticum aestivum* increases the susceptibility of plants to *Puccinia striiformis f.* sp. *tritici*, suggesting that TaCESA7 can restrict lignin biosynthesis (Zhang et al., 2025b). This was evidenced by extensive cell wall lignification, increased activity of phenylalanine ammonia lyase (PAL), upregulation of lignin biosynthesis genes, and stronger accumulation of ROS in infected plants with deficient TaCESA7 activity (Zhang et al., 2025b). Moreover, changes in cellulose synthesis might also affect callose accumulation, as observed in *Xanthomonas campestris*-infected *A. thaliana* (Liu et al., 2024). This bacterial species secretes diffusible signal factor (DSF), which induces CESA and β-1,3-glucanase (BG2), thereby reducing callose abundance in the CW. Infected mutants deficient in the cellulose biosynthesis genes CESA3 (*je5*) and CESA6 (*prc1-1*) exhibited increased callose accumulation and lower ROS accumulation than wild-type (WT) plants, indicating the critical role of certain CESAs in papillae formation (Liu et al., 2024).

### 2.2 Cellulases and pectinases in DAMPs

The next aspect of plant CW modification is cellulose degradation mediated by cellulases (EC 3.2.1.4), which are categorized as cell wall degradation enzymes (CWDEs) (Gibson et al., 2011). This type of enzymes were associated with the pathogen penetration phase (pathogen gaining access to the cytoplasm of plant cells via performing breach in the structure of cell walls). This active incursions through cell wall releasing host peptides and oligosaccharide fragments (de Azevedo Souza et al., 2017). Oligosaccharide fragments are especially important in the case of plant response. The cellulase and pectinases activity induces the formation DAMPs, which include exactly cellulose- and non-cellulose-derived oligosaccharides that initiate a plant defense response (Kesten et al., 2017). The Tanaka and Heil (2021) presented classification of DAMPs as cDAMPs (constitutive DAMPs) and iDAMPs (inducible DAMPs). The cDAMPs are released upon cellular damage, while iDAMPs are actively secreted (as endogenous peptides) upon infections and modulate as host immno signals, also known as phytocytokines (Tanaka and Heil, 2021). The cDAMPs include oligogalacturonide (OGs), eATP, eNAD(P)^+^, and glutamate (Ge et al., 2022). iDAMPs, (known as phytocytokines), are created from precursor proteins (pro-iDAMPs) by proteases during cell wall damage or pathogen infections (Ge et al., 2022). However, one of the best described cDAMPs are oligogalacturonides (OGs). OGs are products of the breakdown of the pectin homogalacturon done by microbial or endogenous pectinases during pathogen infection or cell wall mechanical damage as well as are frequently elucidated in several plant species in wide range of antipathogenic reactions along with accumulation of phytoalexins (de Azevedo Souza et al., 2017) or callose deposition and ROS production (Galletti et al., 2008). Moreover, OGs work via partially the same signaling pathways as PAMPs to elicit defenses and provide protection against pathogens. The OGs signal/presence is recognized by cell wall-associated receptor-like kinases (WAK1 and WAK2). Kohorn et al. (2006) showed that cell wall-associated receptor-like kinases are crucial for proper CW components synthesis and expansion. Other studies outlined that WAK1 carries an N-terminal pectin binding domain that interacts with non-methylesterified HGA and OGs in dependence on Ca^2+^ (Decreux and Messiaenm, 2005). Furthermore, OGs with elicitor activity can bind in reversibly way to WAK1. Strength of binding increased in the case of OGs organized in a calcium-mediated “egg box” conformation (Cabrera et al., 2008; Turella et al., 2025). Decreux and Messiaenm (2005) and Kohorn et al. (2009) postulated biding preference of WAK1 and WAK2 for de-esterified HGA and for OGs. Moreover, Brutus et al. (2010) investigation indicated that WAK1 acts as a receptor of OGs and overexpression of *WAK1* increased resistance to the necrotrophic fungal pathogen *Botrytis cinerea*. OGs affect also plant gene expression as well as OGs treatment can induced expression of more 1000 genes in *Arabidopsis* seedlings (Ferrari et al., 2007). Furthermore, Huerta et al. (2023) outlined also that WAK1/OGs created a pair of interaction DAMPS/PRRs. Other DAMPs interact also with PRRs and create unique DAMPS/PRRs pairs like in the case of: Pep (peptides)/PEP1 RECEPTOR 1 (PEPR1), PEPR2 (Huffaker et al., 2006; Yamaguchi et al., 2006, 2010) and extracellular ATP (eATP)/DOES NOT RESPOND TO NUCLEOTIDES 1 (DORN1) (Choi et al., 2014). Known cDAMPS/PRRs are LecRK-I.9/eATP, LecRK-I.5/eATP, LecRK-I.8./eNAD+ and LecRK-I.8./eNADP+ LecRK VI.2, and GLR3.3 and GLR3.6 with glutamate (Ge et al., 2022). The direct and correct recognition of DAMPs signals by PRRs activates PTI (Tsuda and Katagiri, 2010). During the process, the cellular Ca^2+^ concentration is increased along with synthesis of ROS and activation of MAPK cascades (Zipfel and Robatzek, 2010; Tena et al., 2011; Bi et al., 2018).

Cellobiose is a cellulose-derived cDAMP (different in origin than OGs) consisting of two cellulose units connected by two β-1,4-glycosidic bonds (de Azevedo Souza et al., 2017). Cellobiose activates genes associated with suberin biosynthesis, which enhances resistance to *Pseudomonas syringae* pv. *tomato* DC3000 in *A. thaliana* plants (de Azevedo Souza et al., 2017). The de Azevedo Souza et al. (2017) outlined that Arabidopsis plants are able not only to perceive the presence of cellobiose signal but also activate a signaling cascade leading to the upregulation of defense-related genes, with substantial overlap relative to other pathogen-associated and cell wall damage-associated elicitors. Moreover, the use of cellobiose pretreatment not only mitigated the essential cell wall damage caused by *P. syringae* infection but also generated a rapid and transient intracellular calcium spike, which was similar to the timing and shape of the calcium response to OGs (de Azevedo Souza et al., 2017). It is possible that concurrent DAMPS/PAMP perception led to synergistic changes in Ca^2+^ signaling in raised immune potential. Furthermore, cellobiose activate also MAPKs at early stages of infection without ROS involvement creating MAPK signaling crucial for activation plant immunity (Ranf et al., 2011).

Cellulases are glycoside hydrolases (EC 3.2.1.4) from families GH5, GH6, GH7, and GH45 (Yan and Wu, 2013; Rafiei et al., 2021). Some plant cellulases, for example, endo-β-1,4-glucanases (EGases) (EC 3.2.1.4), are induced during pathogen infection, and their activity leads to the disintegration of β-glucans (Sivaramakrishnan et al., 2024). The endo-β-1,4-glucanase *LrCel1* from *Lilium regale* enhances susceptibility to *Fusarium oxysporum* infection in transgenic *Nicotiana benthamiana* and *Nicotiana tabacum* plants (Chen et al., 2025). *LrCel1* expression was mitigated during the fungal infection (**Table 1**); however, *LrCel1* overexpression in transgenic lines caused reduction in cellulose, lignin, and callose biosynthesis (Chen et al., 2025). The negative impact of EGases on the plant CW can be attenuated by γ-aminobutyric acid (GABA) and its enzyme gamma-aminobutyric acid transaminase (GABA-T) in *Zea mays* cells infected with necrotrophic *Rhizoctonia solani* (Guo et al., 2022). GABA-T causes the degradation of EG1, while GABA suppresses *EG1* expression, resulting in the restriction of ROS generation and mitigation of the necrotic lesion area (Guo et al., 2022). Moreover, the catalytic activity of *Rhizoctonia solani* EG1 is not required to initiate plant responses, as evidenced by the comparison of symptoms between *A. thaliana*, *Z. mays*, and *N. tabacum* plants injected with wild-type or mutated form of EG1 (without catalytic activity) produced in yeast system (Ma et al., 2015). Surprisingly, the results indicated that the molecule without activity is sufficient to stimulate the plant response, and every tested plant exhibited symptoms characteristic of the HR regardless of high or low cellulase activity. According to Ma et al. (2015) EG1 (in wild type/mutated forms) was able to induce cell death in plant with usage the *Potexvirus ecspotati* (PVX) expression system. Further *in vivo* experiments associated with expression of *EG1* suggested it involvement in cell death during infection *R. solani* in maize. It was also outlined that endoglucanase can act as an elicitor, but its enzymatic activity is not required for elicitor activity.

### 2.3 Glycosyltransferases

In addition to glycoside hydrolases (GHs), glycosyltransferases (GTs) are very important enzymes for maintaining CW integrity; they not only participate in CW expansion and plant development but also manipulate plant defensive responses to pathogens (Lyu et al., 2015; Amos and Mohnen, 2019; Zhang et al., 2025a). GTs catalyze the synthesis of xylan—the most abundant plant hemicellulose. Xylan is a linear polysaccharide containing xylose residues connected through β-(1,4)-linkages; it is produced in the membranes of the Golgi apparatus and may be substituted with arabinose, glucuronic acid, or other monosaccharides (Rennie and Scheller, 2014). The primary structure of xylan is formed by three irregular xylem (IRX) enzymes, namely IRX9, IRX14, and IRX10; substitutions include glucuronic acid substitution enzymes of xylan (GUX) and other enzymes (Rennie and Scheller, 2014). IRX refers to structural changes in vascular bundles (caused due to restricted xylan synthesis); these changes were observed using a transmission electron microscope when *IRX* genes were silenced (Brown et al., 2011). Under native conditions, IRXs are co-expressed with cellulose synthases related to the secondary CW formation (Brown et al., 2011). However, the GUX enzyme GhGUX5 was involved in developing resistance (**Table 1**) of *Gossypium hirsutum* to *Verticillium dahliae* (Zhang et al., 2023). Following the inoculation of the pathogen, changes in lignin synthesis upregulated *GhGUX5* expression. In contrast, infected plants not expressing *GhGUX5* showed a major reduction in cell wall lignification and increased susceptibility to *V. dahliae* (Zhang et al., 2023).

Tomato wound-induced 1 (Twi1), another GT from *Solanum lycopersicum*, is an example of uridine diphosphate (UDP) sugar-dependent glycosyltransferases (UGTs) involved in modifying phenolic compounds. *Twi1* was upregulated after inoculation of the plant with a virulent strain of *P. syringae* pv. *tomato* (*Pst*) DC3000 and *Orthotospovirus tomatomaculae* (TSWV) (Campos et al., 2019; Zhang et al., 2025a). Higher Twi1 activity strongly mitigated the HR in transgenic *S. lycopersicum* plants infected with TSWV owing to elevated antioxidant activity that reduced ROS accumulation (Campos et al., 2019). The other UGTs (*UGT73C3* and *UGT73C4*) highly affected plant immunity (**Table 1**) and CW structure of *A. thaliana* plants infected with *P. syringae* pv. *tomato* (*Pst)* DC3000 (Zhao et al., 2025). Substantial CW lignification was observed in knockout double mutants of *UGT73C3 and UGT73C4*. In contrast, WT plants express *UGT73C3 or UGT73C4* more intensively accumulated callose in the CW (Zhao et al., 2025). *CaUGT1*, another UGT from *Capsicum annum*, is highly induced during the HR (**Table 1**; **Supplementary Table 1**) against *Tobamovirus tabaci* (TMV) *and Xanthomonas campestris* pv. *vesicatoria* (Xcv) (Lee et al., 2009). *CaUGT1* silencing caused major delay in HR symptom development and decreased the overall number of HR lesions (Lee et al., 2009). Additionally, in *Linum usitatissimum* inoculated with *F. oxysporum*, the expression of other hemicellulose synthesis enzymes showed changes (Wojtasik et al., 2016). At 48 days post-infection, these changes involved downregulation of hemicellulose synthesis genes, including glucomannan 4-β-mannosyltransferase (GMT, EC 2.4.1.32), galactomannan galactosyltransferase (GGT, EC 2.4.1.38), and xyloglucan xylosyltransferase (XXT, EC 2.4.2.39); gradual upregulation of the degradation enzyme α-galactosidase (GS, EC 3.2.1.22); and sudden induction of endo-β-mannosidase (MS, EC 3.2.1.78). Moreover, the changes in CW structure were associated with a reduction in lignin content and an increase in overall CW plasticity (Wojtasik et al., 2016).

### 2.4 Xyloglucan endotransglycosylase

The hemicellulose fraction of plant CW is also remodeled during biotic stresses (DeTemple and Zabotina, 2025). The most common structural changes in the hemicellulose fraction of plant CW are associated with xyloglucan endotransglycosylase/hydrolase (XTH) (EC 2.4.1.207) (Pauly et al., 2013). These enzymes influence plant response to pathogens (Sarmiento-López et al., 2023) For instance, *xylosidase1* (*XYL1*) from *A. thaliana* is required to maintain the association between cellulose and hemicelluloses in the CW (Günl and Pauly, 2011). Plants with a mutated *XYL1* exhibited a lower concentration of soluble xyloglucan content in the fraction intertwined with cellulose, indicating a different arrangement of XyG domain structure in mutants compared to that in WT plants (Günl and Pauly, 2011). Xyloglucosyl transferase (XTH-Xet5) (E.C. 2.4.1.207) detected in *Solanum tuberosum* during incompatible (**Figure 3**) interactions with PVY^NTN^ was associated with the strongly resistant HR exhibited (**Supplementary Table 1**) by potato cultivar Sárpo Mira (Otulak-Kozieł et al., 2018). The enzyme molecules were particularly localized in the CW of vascular bundles and parenchymal cells of infected leaflets. This positioning contributed to the rebuilding of observable thickened CWs, achieved by reorganizing the network of cellulose and hemicelluloses connected following XTH activity, thereby influencing the physical properties of the CW (Otulak-Kozieł et al., 2018). Conversely, NbXTH from *N. benthamiana* confers susceptibility to TMV infection (Ershova et al., 2025). The fluorescence analysis of the distribution of the GFP vector in transgenic plants revealed that increased *NbXTH* expression is correlated with enhanced local movement of large biomolecules, suggesting that the enzyme influences the plant CW structure around plasmodesmata (Ershova et al., 2025). In susceptible cultivar Valencia Late (VL) of *Citrus × sinensis* infected with *Closterovirus tristezae* (CTV), *endotransglucosylase 9* (*xth9*) was upregulated after persistent high subcellular virus titers 31 months after inoculation (da Silva et al., 2023). XTH9 can also activate MAPK cascades, leading to further CW lignification; however, elevated gene expression significantly contributed to CTV development (da Silva et al., 2023).

### 2.5 Xylanase inhibitors

Interestingly, plant pathogens can induce the production of hostile xylanases to overcome the strengthening of plant CW. The best-known fungal xylanases are BcXyl1 and Xyn11A produced by *Botrytis* (Yang et al., 2018b; Noda et al., 2010) and RcXYN1 from *Rhizoctonia cerealis* (Lu et al., 2020). To counter this response, plants can induce xylanase inhibitors (XIs) (Tundo et al., 2022). In general XIs support increase plant resistance to pathogens. A rice xylanase inhibitor (RIXI) was detected in *Oryza sativa* cv. Nipponbare; this inhibitor contributed to the reduction of virulence of *Magnaporthe oryzae* (Hou et al., 2015). It does not interact with GH10-family xylanases but inhibits the activity of pathogenic GH11 enzymes. *Triticum aestivum* xylanase inhibitor-I (TAXI-I) attenuates BcXyl1 ability to induce necrosis in plant cells (Tundo et al., 2020). Moreover, TAXI-III and xylanase inhibiting protein (XIP-I) combine these two abilities and reduce the catalytic and necrotizing effects of xylanase (Tundo et al., 2015). Furthermore, Tundo et al. (2016) also noted the additive effect of stacking TAXI-III and PvPGIP2 (a PGIP from *Phaseolus vulgaris*) in durum wheat, resulting in enhanced disease resistance against *F. graminearum* compared to parental lines individually carrying TAXI-III or PvPGIP2.

## 3 Extensins and expansins involved in CW modifications induced by plant microbial pathogens

### 3.1 Extensins

Plant defense responses are not only regulated by enzymes but also by cell wall-associated non-enzymatic proteins (CWPs) (Rashid, 2016; Merkouropoulos and Shirsat, 2003). CWPs significantly influence plant CW durability. Two main subfamilies from seven EXTs are glycine-rich proteins (GRPs) and hydroxyproline-rich glycoproteins (HRGPs) (Cheng et al., 2024). The GRP superfamily comprises proteins with a predominance of glycine in the amino acid sequence (up to 70%); these proteins not only show antimicrobial activity, but they also participate in transmitting infection signal to the CW-associated kinases (Czolpinska and Rurek, 2018). The most important HRGPs play a role in forming an intramolecular network of cross-linked proteins (Deepak et al., 2010). Extensins belong to the second group, and they function in strengthening the CW, limiting CW permeability, and facilitating CW lignification (Rashid, 2016). They have -Tyr-X-Tyr- motifs, allowing the formation of a protein network that can strengthen the CW (Mishler-Elmore et al., 2021). The generated protein networks also contribute to the sensing of CW integrity and transmission of mechanical and molecular signals to the membrane and cytoplasmic components (Herger et al., 2019).

An example of extensins (HRGPs) participating in CW (**Supplementary Table 1**; **Figure 3**) strengthening is *StEXT4* from *S. tuberosum* undergoing significant changes in the expression pattern during *Potyvirus yituberosi^NTN^* (PVY^NTN^) infection (Otulak-Kozieł et al., 2020). Elevated *StEXT4* expression levels were detected in the resistant cultivar Sárpo Mira. The HGRPs were mostly localized in the CW during the HR (Otulak-Kozieł et al., 2020). Similarly, in a study conducted on wounded and intact *Musa* spp. plants infected with *F. oxysporum*, immunolocalization analysis of extensins showed their increased accumulation after pathogen contact (Wu et al., 2017). The analysis also revealed that additional extensins labeled with JIM11 were detected in resistant plants, whereas extensins labeled with JIM20 were found in susceptible plants (Wu et al., 2017). JIM11 detects extensins localized in the endodermis, whereas JIM20 is specific for extensins located in the intrafascicular parenchymal region of stem and roots between vascular bundles; this finding suggests that the incompatible interaction of *Musa* spp. with the fungus caused changes in the extensin pool between neighboring cell groups (Hall et al., 2013). Viral infections may also lead to the indirect induction of extensins following lateral root overgeneration. This conclusion is based on the expression analysis of the expansin genes *BvEXLA1a* and *BvEXPA4L* from *Beta vulgaris* infected with *Benyvirus necrobetae* (BNYVV) (Fernando Gil et al., 2018).

Plants also produce molecules similar to extensins. Extensin-like protein (ELP) can influence plant-pathogen interactions (**Table 1**; **Figure 2**) of *S. lycopersicum* with *Clavibacter michiganensis* subsp. *michiganensis* (Cmm) (Balaji and Smart, 2012). ELP overproduction in tomato plant cells delayed the onset of systemic infection symptoms from 12 dpi to 20 dpi, attenuating the pathogenicity of the Cmm strain. This was followed by restriction of necrotic spot development and strengthening of the CW (Balaji and Smart, 2012). Proline extensin-like receptor kinase 1 (PERK1) is another protein with structural similarity to extensins; it anchors the plasma membrane and strengthens the CW (**Table 1**; **Figure 2**) of *Brassica napus* cells infected with *Sclerotinia sclerotiorum* (Silva and Goring, 2002). The PERK1 gene expression level increased after inoculation with *S. sclerotiorum* following fungus-associated CW damage (Silva and Goring, 2002).

### 3.2 Expansins

Expansins participate in relaxing weak noncovalent interactions between cellulose microfibrils and hemicelluloses, leading to CW expansion (Narváez-Barragán et al., 2022). They are classified into two families: α-expansins, which mediate CW degradation induced by a low pH value, and β-expansins, which facilitate the disruption of pectin-cellulose bond in the middle lamella (Cosgrove, 2015). Although the carbohydrate binding sites of expansins show structural similarity to GH family proteins, they do not exhibit enzymatic activities (Cosgrove, 2015). Expansins reduce CW rigidness in *N. benthamiana-*TMV interactions (Chen et al., 2018). Plants with silenced *EXPA4* showed a lower concentration of virus coat protein and viral mRNA and were less vulnerable to wounding compared to WT plants and those with *EXPA4* overexpression (Chen et al., 2018). It was assumed that this gene contributes to the generation of deformations in the CW, facilitating local transport of the virus (Chen et al., 2018). The expression level of *NbEXPA1*, another expansin gene, was altered in *N. benthamiana* cells infected with *Potyvirus rapae* (TuMV) (Park et al., 2017). The authors reported that EXPA1 is involved in cell-to-cell viral transport (**Figure 3**), as the protein is located in the CW surrounding plasmodesmata and at viral replication sites, suggesting its critical role in viral replication (Park et al., 2017). However, *NbEXPA1* expression was downregulated to 60% in inoculated leaves compared to that in the control sample; thus, the downregulation of expression appears to a component of the plant’s mechanism to prevent local migration of the virus (Park et al., 2017).

On the other hand, expansins are also produced by microorganisms interacting with plants, including bacterial crop pests, oomycetes, and fungi that allow them to colonize the xylem by changing the plant CW structure (Cosgrove, 2017; Narváez-Barragán et al., 2020; Nikolaidis et al., 2014).

## 4 Involvement of pectin methylesterases and their inhibitors in CW modifications induced by pathogenic microorganisms

CW reorganization can also occur through enzymatic modification (**Supplementary Table 1**) in the CW pectin fraction (Haas et al., 2021; Voragen et al., 2009). Homogalacturonan (HG) is the most abundant and important pectin in plants; it consists of unbranched galacturonic acid chains modified by a wide group of molecules collectively called HG-modifying enzymes (HGMEs) (Sénéchal et al., 2014). The most important HGME is pectin methylesterase (PME; EC 3.1.1.11), which catalyzes HG demethylation; this process is regulated by pectin methylesterase inhibitors (PMEIs) (Wormit and Usadel, 2018). PME affects CW structure and facilitates the decomposition of CW pectins (Francocci et al., 2013). However, multiple plant pathogens can induce plant’s PME to increase CW permeability, leading to PMEI induction as the plant response. In *A. thaliana* cells infected with *Pectobacterium carotovorum*, the bacteria stimulated (**Figure 1**) the expression of the plant methylesterase homolog *AtPME3*, which restricted pectin methylesterification and led to severe infection symptoms (Raiola et al., 2011). In the *Atpme3* knockout mutants, the degree of pectin methylesterification (DM) was higher than that in control plants, resulting in mild infection symptoms. *Arabidopsis* mutants overexpressing *AtPMEI-1 and AtPMEI-2* showed the least damage to leaf lamina and lower bacteria concentration (Raiola et al., 2011). The induction of PMEI genes, detected by RNA *in situ* localization with similar observations, was also found in *Capsicum annuum* inoculated with *Xanthomonas campestris* pv. *vesicatoria* (An et al., 2008).

A genome screening research study revealed the importance of PMEI induction in the resistance (R-line) plants of *Brassica napus* infected with *S. sclerotiorum*; the intensively induced *BnPMEI127* and *BnPMEI76* were identified as the most resistance-related gene (Wang et al., 2022). In another study on *PMEI11-*overexpressing *A. thaliana* plants inoculated with *B. cinereal*, the reduction in PME3 activity highly mitigated fungal colonization (Lionetti et al., 2007). Infection with another fungus *B. cinerea* increased *AtPME3* expression in *A. thaliana* (Raiola et al., 2011). In WT plants, *AtPME3* was significantly upregulated after 72 hpi, whereas the expression was mitigated in the homozygous *pme3* mutant, which reduced the overall PME activity by half compared to that in the WT plant. This increased the abundance of highly methylated pectins but did not significantly change CW monosaccharide profile and had an inconsequential impact on plant-fungus interaction (Raiola et al., 2011). A similar reduction in GhPME2 and GhPME31 accumulation was observed in the WT plants of *G. hirsutum*; *GhPMEI3* induction occurred in response to *V. dahlia* infection, resulting in an increase in CW pectin DM compared to that in knockout mutants (Liu et al., 2018). This upregulation of *GhPMEI3* was stimulated by oligogalacturonides (OGs) released from the CW, during the development of invasive hyphae (Liu et al., 2018). OGs formed after polysaccharide degradation and activate a general defense response to biotic threats (Ferrari et al., 2013). *V. dahliae* also produces VdPME1, which actively interacts with moderately methylesterified pectins, and polygalacturonase VdPG2, which supports the function of VdPME1 by cleaving bonds between non-methylesterified pectin residues, thereby increasing pectin accessibility to enzymatic cleavage (Safran et al., 2021). CW modification induced by *V. dahliae* involves the generation of OGs, which might be an important signal transducer influencing plant-pathogen interactions. Interestingly, susceptible cultivar cells were lacking GalA3Me oligosaccharides, which were otherwise present in the resistant cultivar; this finding suggests that particular OG might be vital for activating defense response (Safran et al., 2021).

In plant-virus interactions (**Figure 3**), PME activity was reduced in transgenic *N. tabacum* plants (**Table 1**) infected with TMV and overexpressing *Actinidia chinensis* PMEI *(AcPMEI*) (Lionetti et al., 2014). Overall, PME activity was decreased compared to that in the control plants, accompanied by an increase in CW pectin DM (Lionetti et al., 2014). In *A. thaliana* respiratory burst oxidase homologs knockout mutants (*rbohD/F*) inoculated with TuMV, AtPMEI-2 and AtPMEI-3 inhibitors successfully suppressed PME activity (Otulak-Kozieł et al., 2024). *AtPMEI2* and *AtPMEI3* were highly upregulated during the resistance response, accompanied by *AtPME3* downregulation. Moreover, the accumulation of PMEIs drastically strengthened the CW and diminished PME activity in *rbohD/F* plants (Otulak-Kozieł et al., 2024). In contrast, PME accumulation or activity did not increase in the response of *A. thaliana* plants overexpressing *AtPMEI-1* and *AtPMEI-2* to *Tobamovirus rapae* (TVCV) (Lionetti et al., 2014).

## 5 Involvement of PR proteins in CW modifications induced by plant pathogens

Plants have developed multiple PR protein families that play a role in overall antimicrobial defense and CW structural alterations (Ebrahim et al., 2011; Elvira et al., 2008). PR proteins with enzymatic activity can act extracellularly in the apoplast in response to pathogen infection (**Supplementary Table 1**), particularly in local CW thickenings (Martínez-González et al., 2018; Jain and Khurana, 2018; dos Santos and Franco, 2023). They can also activate host defense mechanisms related to CW rearrangement and participate in macromolecular cross-linking (dos Santos and Franco, 2023; Jain and Khurana, 2018).

PR proteins are classified into 17 distinct classes (PR-1 to PR-17) and occur in most plants (Han et al., 2023; Yang et al., 2018a). Frequently plants have multiple genes usually encode each PR protein family enabling the synthesis of diverse protein isoforms (Han and Schneiter, 2024). PRs include those with enzymatic activity capable of modifying CW structure. PR-3, PR-4, PR-8, and PR-11 are classified as chitinases that can degrade chitin during plant-fungus interactions (Islam et al., 2023). The downregulated distribution or synthesis of PRs with chitinases activity blocks or limits the ability to digestion of fungal pathogens cell and pathogen recognition and antifungal response (Islam et al., 2023). PR-2 shows a β-1,3-glucanase activity and can degrade CW glucans (Chouhan et al., 2023; Yadav et al 2023). Overexpression of *PR-2* showed that PR-2 negatively affects callose deposition and have promoting effects on infection of *Leptosphaeria maculans* (Oide et al., 2013). The disturbance of callose deposition also can influence the induction of viral infections of TMV (Aseel et al., 2023). PR-5 exhibits various functions such as glucan degradation, xylanase inhibition, and polysaccharide binding (Liu et al., 2010). The overexpression of *PdPR5–1* from *Prunus domestica* in *A.thaliana* enhanced resistance to *Alternaria brassicicola* not only by changes in cell wall but also by induction of phytoalexin (El-kereamy et al., 2011). Interestingly, the induction of enzymatic PR proteins is potentially associated with cell wall lignification. Elevated levels of PR-1, PR-2, PR-3, and PR-5 were detected in *Daucus carota* L. cells overexpressing *OsPRx114*, a class III peroxidase classified as PR-9 (Wally and Punja, 2010). The relative gene expression of *PR-2* and *PR-5* genes increased in bio-primed (pre-inoculated with growth-stimulating fungi *Trichoderma asperellum* and *T. harzianum*) seeds and seedlings of *C. annuum.* Enzymatic pathogenesis-related proteins belonging to the PR-3 group are highly associated with the antifungal response (Chouhan et al., 2023). PR-3 can suppress plant CW degradation during oxidative burst and was highly expressed in the rhizodermis of *G. hirsutum* roots challenged with *F. oxysporum* f. sp. *vasinfectum* (Zambounis et al., 2012). PR-3 activity can also generate chitin oligosaccharides as byproducts released from pathogen CWs (Bacete et al., 2018). The molecular signal from chitin oligosaccharides is transduced by leucine-rich repeat protein receptor kinases (LRR-RK), which are essential for the recognition of microbial-associated molecular patterns (MAMPs) (Fernández-Calvo et al., 2024; Narváez-Barragán et al., 2022). Chitin oligosaccharides trigger the plant defense response by stimulating the production of phytoalexins, CAZymes, and antioxidants as a part of induced systemic resistance (ISR) (Saberi Riseh et al., 2024). Moreover, chitinase accumulation in fungal-infected plant cells is often followed by PR-2 and PR-4 localization, which can bind to chitin (Han and Schneiter, 2024). These findings indicate that plant interactions with pathogens involve the induction of a wide PR protein palette and other molecules rather than a singular protein.

## 6 Conclusions

The plant CW is generally considered a barrier between the content of living cells and the extracellular environment. However, previous studies have shown that the CW dynamically changes during interactions with microorganisms, and the action of the CW proteasome plant may reveal how plants respond to pathogens. The plant CW contains multiple enzymatic and non-enzymatic proteins, whose presence contributes to the modulation of plant responses of susceptible and resistant plants to pathogenic microorganisms. Among these proteins, polysaccharide-modifying enzymes and CWPs play a critical role in altering the response of plants to pathogens. Although these changes can go beyond the immediate effects of their function, the results depend on the individual properties of the plant and the pathogen. Plant CW components are involved in multilevel interactions with other factors participating in the immune response. Hence, establishing a coherent, detailed universal mechanism of plant immunity against microorganisms is a challenging issue. This is even more difficult if we considering that actual research defined several knowledge gaps/bottlenecks in our understanding of cell wall response which seems to be new focus points for research:

(*) Firstly, we do not adequate understanding the roles of particular isoform-specific CESA complexes in cell wall dynamics during plant defense (Kumar et al., 2018). As was suggested by Kumar et al. (2018) primary cell wall requires various subunits of CESAs (1,2,3,5,6 and 9) to form the functional heterotrimeric CSC but CESAs individual roles shift significantly during HR (from mitigation cellulose synthesis to induction of callose deposition upon infection sites). Moreover, we still do not understand why downregulation of some CESAs like: CESA3 or CESA6 directly frees up plasma membrane microdomains, lipid rafts, or substrate pools (UDP-glucose) for callose synthases like PMR4/GSL5 (Desprez et al., 2007). The disruption of CESA3 triggers a massive, constitutive activation of defense pathways, including JA and ethylene production. Whereas loss of function CESA3 leads rather to hyper-accumulation of callose (Desprez et al., 2007). Conversely, disrupting CESA6 generates milder and structurally localized defense. Hu et al. (2016) outlined also that CESA3 strongly activate THE1-dependent signaling pathways, driving to a ROS burst and cell death. On the other hand, CESA6 alterations are frequently associated with parallel or attenuated sensor networks (Hu et al., 2016). This “paradox” created blurs in the border line between structural cell wall defects and a targeted immune signaling cascade (Hu et al., 2016).

(**) The XI-xylanase complexes gaps described as “stoichiometric dilemma” and “spatial conflict”. The “stoichiometric dilemma” indicates unclear role of XIs modulation of HR amplitude simply by physically burying the xylanase epitopes required for receptor recognition, or if a specific threshold ratio of unbound/bound enzyme dictates the strength of the cell-death signal (Flatman et al., 2002; Fierens et al., 2007; Tundo et al., 2022; Zhao et al., 2024). The “spatial conflict” is ambiguous whether XIs strictly exert their HR-modulating effects in the extracellular matrix by preventing cell-wall damage (which yields damage-associated molecular patterns), or if internal transport mechanisms funnel complexed components into the cytosol to alter internal plant host stress surveillance system (Tundo et al., 2022; Zhao et al., 2024).

(***) The gap associated with existence of microbial expansins (EXLXs) which is non-lytic proteins homologous to plant expansins (Narváez-Barragán et al., 2020; Haddad Momeni et al., 2024). Pathogens often express EXLXs locally at the direct contact zones between the pathogen (e.g., fungal appressoria or bacterial infection threads) and plant host wall in a very narrow timeframes—specifically during initial penetration stage (Morales-Quintana et al., 2021; Pohto et al., 2026). Their activity relies on non-enzymatic disruption of hydrogen bonds between cellulose and hemicellulose, EXLXs do not leave behind stable, easily quantifiable chemical footprints (Pohto et al., 2026). Therefore, EXLXs tracing requires advanced live-cell imaging that can resolve events occurring within nanoscale wall layers to show changes during infection (Morales-Quintana et al., 2021). These facts suggest that spatial-temporal activity, host target specificity, and physiological impact on CW integrity during infection in case of EXLXs are largely unknown.

A thorough understanding and explanation of knowledge gaps/bottlenecks will require multiple complete datasets from various pathosystems with possible development of new research methods. In this context is needed also to formulate short and long terms focus points. Among short term we could outline CRISPR-based isoform-resolved CESA mutagenesis in various model host or high-resolution imaging of XI localization during HR. In the long-term approaches engineering XI- or expansin-inspired biotechnologies will be crucial for creation durable crop resistance breeding programs. Overall future research should focus not only on multileveled and complex approaches to cell wall remodeling in reaction against pathogens with use of quantity and quality assessments (by capabilities of biochemical and biophysical analyses) of CW properties but also with integrative use of active computer or AI-developing modeling during pathogen incursion in plant host. Moreover, this potentially new research findings and model approach also facilitate the development of resistant cultivars/durable crop resistance programs and could serve as a basis for designing current trends in plant protection strategies but also to creation of maybe long term “reactive models” for more rapid progress in “arms race” between pathogens and host in favor of plants. Thus, CW enzymes and proteins appear to have incredibly promising potential for future research investigations and modeling in plant interactions with pathogenic microorganisms.
