# Supplementary material for: Selected cell wall-associated components in plant defense responses against microbial pathogens
Source: Front Plant Sci. 2026 Jul 14;17:1896272. doi: 10.3389/fpls.2026.1896272 (PMC13408270; doi:10.3389/fpls.2026.1896272)
Supplement: Supplementary file 1 [file Table1.docx]

**Supplementary Table 1.** The involvement of CW-associated proteins and their genes in resistant reactions against bacteria, viruses and fungi.

| **Type** | **Gene** | **NCBI ID of the gene/genes** | **Protein** | **Protein(s)Uniprot primary accession no.** | **Function** | **Associated pathosystem(s)** | **Reference(s)** |
| --- | --- | --- | --- | --- | --- | --- | --- |
| **Plant resistant reactions against bacteria** | | | | | | | |
| PR proteins | *AtPR1b* | 815945 | AtPR1 | Q9ZNS4 | The peptide CAPE1, released from PR1 after proteolytic cleavage, induces the expression of *PR-1b, PR-2*, *PR-7* in HR and other resistance reactions. | *Arabidopsis thaliana- Pseudomonas syringae* DC3000 | Chen et al., 2014 |
|  | *NtPR1a* | 142181287 | NtPR1a | P08299 | The overexpressed gene participates in HR activation, induces the expression of *Nicotiana tabacum* HR-related genes: *NtHSR201* (hypersensitive -related 201), *NtHIN1* (harpin induced 1), *NtPR2*, NtCHN50 (endochitinase B 50), *NtPR1b*, *NtEFE26* (ethylene-forming enzyme 26), *NtGST1* (Glutathione S-transferase 1). | *Nicotiana tabacum* (transgenic)- *Ralstonia solanacearum* | Liu et al., 2019 |
|  | *CsPrx25* | unknown | CsPRx25 | unknown | The protein increases sensitivity to HR, ROS production, and cell wall lignification. | *Citrus sinensis -Xanthomonas citri subsp. citri; Citrus madurensis - Xanthomonas citri subsp. citri* | Li et al., 2020 |
|  | *PR-10* | 107864568 | PR10 | A0A1U8GEH7 | The protein regulates HR activation by interaction with LRR proteins. | *Nicotiana benthamiana- Xanthomonas campestris pv vesicatoria* | Choi et al., 2012 |
| extensins | *atExt1* | 844028 | Extensin-1 | Q38913 | The gene helps to retain bacteria within the infection site, and in overexpressing plant cells, its transcripts are detectable at the margins of the necrotic spot. | *Arabidopsis thaliana*- *Xanthomonas campestris pv. campestris; Arabidopsis thaliana- Pseudomonas syringae* | Merkouropoulos & Shirsat, 2003; Wei & Shirsat, 2006 |
| UDP-glucosyltransferase | *CaUGT1* | 107848707 | CaUGT1 | C6ZRH7 | Enzyme influences the development of HR lesions. | *Capsicum annuum- Xanthomonas campestris pv. vesicatoria (Xcv)* | Lee et al., 2009 |
| **Plant resistant reactions against viruses** | | | | | | | |
| PR proteins | *CsPR-1b and CsPR-2* | unknown (*CsPR-1b*)  unknown (*CsPR-2*) | CsPR1  CsPR2 | unknown (CsPR-1b)  unknown (CsPR2) | Proteins accumulate in cells of resistant plants during HR. | *Capsicum chinenese* - *Tobamovirus capsici* spanish strain (PMMoV-S) | Elvira et al., 2008 |
|  | *CaGLP1* | 107839763 | CaGLP1 (PR-16) | A0A2G2XXI8 | The protein localizes in the cell wall during HR. | *Capsicum annuum* - *Tobamovirus tabaci* (TMV) | Park et al., 2004 |
|  | *CcPR-4b* | unknown | CcPR-4 | Q75QH1 | The gene is upregulated during HR, and the protein exhibits weak DNase and RNase activities against viruses while acting in the apoplast. | *Capsicum chinenese* - *Tobamovirus capsici* spanish strain (PMMoV-S) | Guevara-Morato et al., 2010 |
|  | *AtPMEI-2* and *AtPMEI-3* | 820981 (AtPMEI2)  832197 (AtPMEI3) | AtPMEI2  AtPMEI3 | Q9LUV1 (AtPMEI2)  Q84WE4  (AtPMEI3) | Genes are upregulated during HR, reduce pectin demethylesterification, and support cell wall rebuilding. | *Arabidopsis thaliana*- *Potyvirus rapae* (TuMV) | Otulak-Kozieł et al., 2024 |
| **Plant resistant reactions against fungi** | | | | | | | |
| PR proteins | *MdPR10-1*  and *MdPR10-2* | unknown (MdPR10-1)  unknown (MdPR10-2) | MdPR10 | unknown (MdPR10-1)  unknown (MdPR10-2) | Proteins regulate HR development by interacting with LRR proteins. | *Malus Domestica- Alternaria alternate f. sp. Mali* | Zhang et al., 2021 |
|  | *AtPR1* | 815949 | AtPR1 | P33154 | The overexpressed gene enhances plant resistance during HR-like response. | *Nicotiana benthamiana* (transgenic)- *Sclerotinia sclerotiorum* | Han et al., 2023; Yang et al., 2018a |
